# Structure analysis of deleterious nsSNPs in human PALB2 protein for functional inference 

**DOI:** 10.6026/97320630017424

**Published:** 2021-03-31

**Authors:** Noshin Nawar, Anik Paul, Hamida Nooreen Mahmood, Md Ismail Faisal, Md Ismail Hosen, Hossain Uddin Shekhar

**Affiliations:** 1 Clinical Biochemistry and Translational Medicine Laboratory, Department of Biochemistry and Molecular Biology, University of Dhaka, Bangladesh

**Keywords:** PALB2, nsSNP, UTR, in silico characterization

## Abstract

Partner and Localizer of BRCA2 or PALB2 is a typical tumor suppressor protein, that responds to DNA double stranded breaks through homologous recombination repair. Heterozygous mutations in PALB2 are known to contribute to the susceptibility of breast and ovarian cancer. However, there is no comprehensive study characterizing the structural and functional impacts of SNPs located in the PALB2 gene. Therefore, it is of interest to document a comprehensive analysis of coding and non-coding SNPs located at the PALB2 loci using in silico tools. The data for 1455 non-synonymous SNPs (nsSNPs) located in the PALB2 loci were retrieved from the dbSNP database. Comprehensive characterization of the SNPs using a combination of in silico tools such as SIFT, PROVEAN, PolyPhen, PANTHER, PhD-SNP, Pmut, MutPred 2.0 and SNAP-2, identified 28 functionally important SNPs. Among these, 16 nsSNPs were further selected for structural analysis using conservation profile and protein stability. The most deleterious nsSNPs were documented within the WD40 domain of PALB2. A general outline of the structural consequences of each variant was developed using the HOPE project data. These 16 mutant structures were further modelled using SWISS Model and three most damaging mutant models (rs78179744, rs180177123 and rs45525135) were identified. The non-coding SNPs in the 3' UTR region of the PALB2 gene were analyzed for altered miRNA target sites. The comprehensive characterization of the coding and non-coding SNPs in the PALB2 locus has provided a list of damaging SNPs with potential disease association. Further validation through genetic association study will reveal their clinical significance.

## Background

PALB2, a 1186 amino acid residue protein, (the gene for PALB2 is located on chromosome 16p 12.2) is mainly responsible for the co-adjuvancy of BRCA1 and BRCA2 in the DNA damage response pathway [1]. PALB2 was first identified as a BRCA2 interacting protein for recruiting BRCA2 to DNA damage repair sites. Later its contribution to tumor suppression was also recognized [2]. PALB2 consists of several protein domains including N-terminal coiled- coil domain- that interacts with BRCA1, C terminal WD40 domain-that interacts with BRCA2 and Chromatin Association Motif (ChAM)-that promotes chromatin association [3]. Impairment of N-terminal coiled-coil domain-BRCA1interaction is associated with breast cancer risk [4]. Whereas, a single nucleotide change in PALB2 C-terminal domain can disrupt its interaction with BRCA2 [5]. As it directly interacts with BRCA2, it has significant role in participating in homologous recombination repair [6-7]. PALB2 is not only responsible for recruiting BRCA2 but also it physically interacts with RAD51 and stimulates D-loop formation [8]. It has a cooperative effect on RAD51AP1, an enhancer of RAD51. With RAD51AP1, PALB2 synergistically stimulates D-loop formation by RAD51 mediated strand exchange. Hence, mutation of these damage repair proteins would lead to impaired DNA repair mechanism and eventually might result in cancer development [9-10]. Heterozygous mutation of BRCA1 and BRCA2 genes along with PALB2 contribute to high risk female breast cancer and ovarian cancer [11]. Several studies including breast cancer patients from Russia, Germany and northwest region of China have been identified with PALB2 mutations [12-13]. Single nucleotide polymorphisms (SNPs) are responsible for increased/decreased susceptibility to certain diseases and thus allowing researchers to evaluate a person's genetic predisposition in developing disease[14]. Recently a combination of SNP and GWAS study has been proved to be useful in determining biological markers for diagnosis of disease [15]. Non-synonymous SNPs consist of a group of SNPs that alters the amino acid sequence of a protein affecting the phenotype. Thus mutation in an non-synonymous SNP can have deleterious impact on the protein activity [16]. Bioinformatics tools are now developed to classify these damaging non- synonymous SNPs as well as to determine their level of pathogenicity [17]. It is of interest to document the analysis of non-synonymous SNPs of PALB2 due to the importance of PALB2 in DNA damage response and its contribution to developing breast cancer risk.

## Methodology

**Figure 1:**
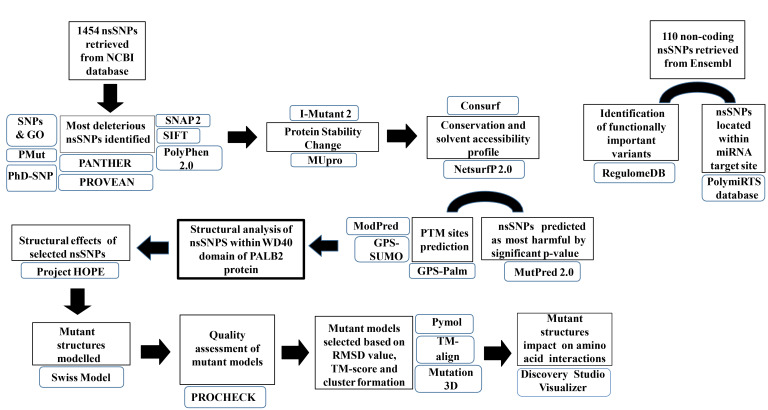
Workflow of nsSNP analysis.

The overall flowchart of the major steps followed in this study is demonstrated via a generalized workflow (Figure 1).

## Retrieval of nsSNPs:

The nsSNPs of the human PALB2 were retrieved from NCBI (National center for Biotechnology Information) dbSNP database [18]. This was refined using non-synonymous in the function class category as a filter. The rsIDs of the nsSNPs were collected for further computational survey.

## Prediction of Deleterious nsSNPs:

For distinguishing the deleterious nsSNPs from tolerated ones, total eight bioinformatics tools were implemented. These tools include: SIFT, PolyPhen 2.0, PROVEAN, PANTHER, SNAP2, PhD-SNP, SNPs and GO, and PMut. The rsIDs of the nsSNPs were provided as input in the SIFT (Sorting Intolerant from Tolerant) tool [19]. NsSNPs that showed probability score less than 0.05 were considered as deleterious and score equal or above 0.05 were considered as tolerated. Further functional characterization was performed using the Polyphen 2.0 (Polymorphism Phenotyping v2), tool [20]. For this purpose, the FASTA format of protein sequence with specific substitutions were submitted to the Polyphen 2.0 tool and a position specific independent count (PSIC) rendering 0.0 as tolerated, 0.801-1.0 as probably damaging and 1.0 as deleterious was generated [21]. For analyzing the effects of the nsSNPs, PROVEAN (Protein Variation Effect Analyzer) tool was used [22]. Similar to the Polyphen 2.0 tool, the PROVEAN uses the FASTA format of the protein sequence along with amino acid substitutions as input query. The output is provided as deleterious or neutral based on the predicted scores (A score below or equal -2.5 were considered as "deleterious" and above -2.5 as neutral). The plain protein sequence and the amino acid variants were submitted to the PANTHER (Protein Analysis Through Evolutionary Relationships) tool. PANTHER provides output in the form of the approximate length of time (in millions of years) for a given amino acid to be preserved. The longer the preservation time the more likely it causes a deleterious effect. After that, analysis of the nsSNPs using the SNAP2.0 (Screening for Non-Acceptable Polymorphism v2), a classifier based on machine learning device called Neural Network was utilized to predict disease mutations [23]. Protein sequence in FASTA format was placed as the input query. SNAP2 score interpretation relied upon the following threshold: neutral: -100 ≤ SNAP2 score≤ 0 and effect: 0< SNAP2 score≤ 100 [24]. Further analysis was carried out using the PhD-SNP (Predictor of human Deleterious Single Nucleotide Polymorphisms), a multiple sequence based alignment tool to predict whether a new phenotype derived from a nsSNP can be related to genetic disease in humans [25]. SNPs and GO (Single Nucleotide Polymorphisms and Gene Ontology) was used to predict the amino acid variations associated with the emergence of diseases in humans [26]. The input query for this was the FASTA format of the protein sequence along with the amino acid variations. Finally, the PMut tool was implemented for the annotation of pathological mutations on protein by Neural Network-based classifier. The predicted scores between 0 and 0.5 were considered as neutral whereas, scores between 0.5 and 1.0 were seen as pathological [27].

## Impact of nsSNPs on protein stability:

Protein stability alterations due to SNPs were measured via two web-based tools. They were support vector machine-based tools named I-Mutant 2.0 and MUpro. Here, the protein sequence collected from NCBI database was provided as input to predict the direction towards which the mutation causes the protein stability change, expressed in DDG value. Positive DDG value indicates increased protein stability, and negative DDG value, protein destabilization [28]. MUpro based protein stability alteration measurement was similar to I-Mutant 2.0, inferring output by the sign of DDG value. Plain protein sequence, mutation position and variant were submitted as query.

## Prediction of conserved residue:

Consurf was used to predict the conservancy of the residues at the SNP positions. The FASTA format of the protein sequence was provided as input in the Consurf tool and the output was provided in a score scheme starting from 1 to 9 with colour differences following Bayesian calculation method. Score 9 with deep purple colour represented highly conserved residue, whereas score 1 with deep blue colour represented a highly variable residue.

## Prediction of surface and solvent accessibility:

Prediction of the secondary structure, solvent and surface accessibility of a protein was performed using the NetsurfP2.0 [29]. The FASTA format of the protein sequence was provided as input for the prediction.

## Prediction of Post Translational Modification sites:

Posttranslational modification sites were predicted using ModPred. The input was FASTA format of PALB2 and result showed overall PTM sites available in the residue of this protein [30]. Different PTM site predictors such as GPS-SUMO, GPS-Palm, GPS-MSP and iGPS were also explored for the prediction of sumoylation, palmitoylation, methylation and phosphorylation respectively [31].

## MutPred2.0:

It is used to combine genetic and molecular data for interpreting g-score (general score) ranging from 0 to 1, reasoning the probability of structural and functional alteration due to nsSNP MutPred2.0 also yielded the p-value of every alteration depicting clinically significant variants [32]. The score ranged from 0 to 1. Scores that is closer to 1 indicated greater propensity to be pathogenic. Protein sequence in FASTA format and amino acid variations were provided as query.

## Identification of SNPs in non-coding regions:

The amino acid variants located on the UTR regions and microRNA target sites were identified by utilizing Ensembl [18], RegulomeDB and PolymiRTS database.

## Identification of 3' and 5'UTR regions:

Ensembl is a single point of access to an noted genomes for mainly vertebrate species [33]. The amino acid variants (rsIDs) on the UTR regions of PALB2 (transcript id, ENST00000261584.9) were retrieved from Ensembl for further analysis by RegulomeDB. RegulomeDB demonstrates whether the variant has any potential functional consequences on the gene regulation [34] The SNPs in the UTR collected from Ensembl were submitted and each variant was categorized according to its functional confidence.

## Identification of DNA variants in miRNA target sites:

PolymiRTS (Polymorphism in micro RNA Target Site) database was utilized for identification of DNA polymorphisms in miRNA target sites as well as seed regions [35]. DNA variants, responsible for any target site creation or disruption in miRNA seed regions are predicted by this database.

## Structural Analysis:

The WD40 domain of PALB2 protein that has been reported in breast cancer patients was emphasized for structural analysis.

## Project HOPE:

HOPE (Have (y) Our Protein Explained), a web application tool, was utilized for analysis of the difference between wild type and mutant structures. It shows structural and functional consequences upon point mutations such as alterations in amino acid bindings, hydrophobicity changes, charge differences etc. [36]. The FASTA format of the protein sequence was submitted as the input query. The mutated residue of the coding regions was selected each time to illustrate its effects at molecular level.

## Modelling of mutant 3D structures:

Homology modelling and structural validation of the WD40 domain of PALB2 protein regarding the mutant residues were performed by Swiss Model [37]. Upon submitting the mutated sequence of PALB2 WD40 domain, proper template selection was carried out based on several factors such as, coverage, GMQE (Global Model Quality Estimation), sequence identity and resolution. According to QMEAN score, the best quality model was selected. The less the QMEAN score was deviated from 1, the better the model quality [38].

## Quality Assessment of the mutant models:

PROCHECK was used for the overall structural "quality" assessment of the mutant structures modelled by SWISS. PROCHECK generates Ramachandran plot which determines the backbone confirmation using phi/psi dihedral angles [39]. It revealed the distribution of residues in favored, allowed, and disallowed regions.

## RMSD value prediction:

The PyMOL (PyMOL Molecular Graphics System, Version 1.2r3pre, Schrodinger, LLC.) molecular Graphics System, version 2.0 Schrodinger, LLC was implemented for RMSD (Root Mean Square Deviation) score prediction. Superimposition followed by alignment between the wild type and mutant structures were executed. Since RMSD calculates the distance between residue pairs equally, the higher the RMSD value the greater the deviation of the mutant structure from the native one.

## TM score prediction:

TM-Align (Template Modeling -Align) optimizes residue to residue alignment in a sequence independent manner between two different structure of proteins for TM-score (Template Modelling Score) prediction [40]. Tm-align produced result between 0 and 1. TM- score 1 referred to no difference between wild type and the given mutant structure. The more TM score is closer to 0, the higher the deviation. The PDB files of wild type and mutant type structures were submitted as input query.

## Mutation 3D cluster prediction:

Mutation 3D helps to visualize any cluster formation due to the provided amino acid substitutions causing somatic cancer mutations [41]. Gene symbol and the amino acid variants were submitted for prediction. Significance of each cluster was verified by its p-value.

## Discovery studio visualization: Discovery studio visualize:

(Dassault Systems BIOVIA, Discovery Studio Modeling Environment, Release 2017, San Diego: Dassault Systems, 2016) was utilized in case of selected mutant models for deeper insight into their properties. Alterations in hydrogen bond formation, hydrogen bond number was observed after superimposing the wild type and mutant structures. Also, changes in the interaction of the wild type residue and mutated residues with other amino acids were visualized with the help of this program.

## Results:

### Retrieval of nsSNPs:

Total 11725 SNPs were found in Human PALB2 gene from dbSNP database of NCBI. Out of 11725 SNP, 1455 were non-synonymous, 592 were synonymous. The non-synonymous SNPs accounted for 13% of the total SNPs reported in human PALB2 gene.

### Deleterious nsSNPs prediction:

Eight different web-based tools SIFT, PolyPhen2.0, PROVEAN, SNAP2, PhD-SNP, PANTHER, SNPs and GO and PMut were utilized for the prediction of damaging and disease associated nsSNP (Supplementary Table 1).

### SIFT:

SIFT predicts the effects of nsSNPs. Among 1454 nsSNPs, 192 variants were found on SIFT server. Protein coding transcript, Protein ID (ENSP00000261584) was selected for analysis as this transcript ID had the highest SIFT score. Out of 171 variants of this particular transcript, 59 variants were predicted to be deleterious and the remaining 112 as tolerant. The rest of the nsSNPs predictor programs further examined these 171 variants.

### PolyPhen 2.0:

Out of 171 variants, 60 amino acid variants were predicted to be probably damaging, 13 variants were predicted to be possibly damaging and the remaining were predicted as benign. 43% of the total nsSNPs were identified for encouraging possible damaging impact with 57% having no significant effect on PALB2 protein.

### Provean:

PROVEAN is used to identify functionally important nsSNPs. PROVEAN characterized 70 nsSNPs out of 171 as deleterious with a threshold value below -2.5. 41% nsSNPs were identified to have deleterious effect on PALB2 protein.

### Panther:

PANTHER calculates the functional effect caused by a potentially pathogenic or deleterious nsSNP on the protein. It showed the preservation time of 8 nsSNPs to be higher than 450 my (millions of years), 40 nsSNPs to be in between 200my to 400 my and the rest to be below 200my. Thus estimated 28% nsSNPs are probably deleterious and 72% are probably benign.

### SNAP2:

SNAP2 speculates disease-inducing mutations caused by the amino acid variants. It predicted 71 amino acid variants to be damaging. So, 42% nsSNPs were responsible for possible damaging impact on PALB2.

### PhD-SNP: 

PhD-SNP predicted 45 nsSNPs responsible to cause disease in humans. PhD-SNP provided an output with a score between 0 and 1, where, any nsSNP having score greater than 0.5 was considered as pathogenic.

### SNPs and GO:

SNPs and GO predicts whether a variant is disease inducing or not. It provided results estimating 21nsSNPs out of 171nsSNPs to be associated with insurgence of disease in humans. Thus, 12% nsSNPs were predicted to be deleterious with the rest having no noteworthy effect.

### PMut:

**Figure 2:**
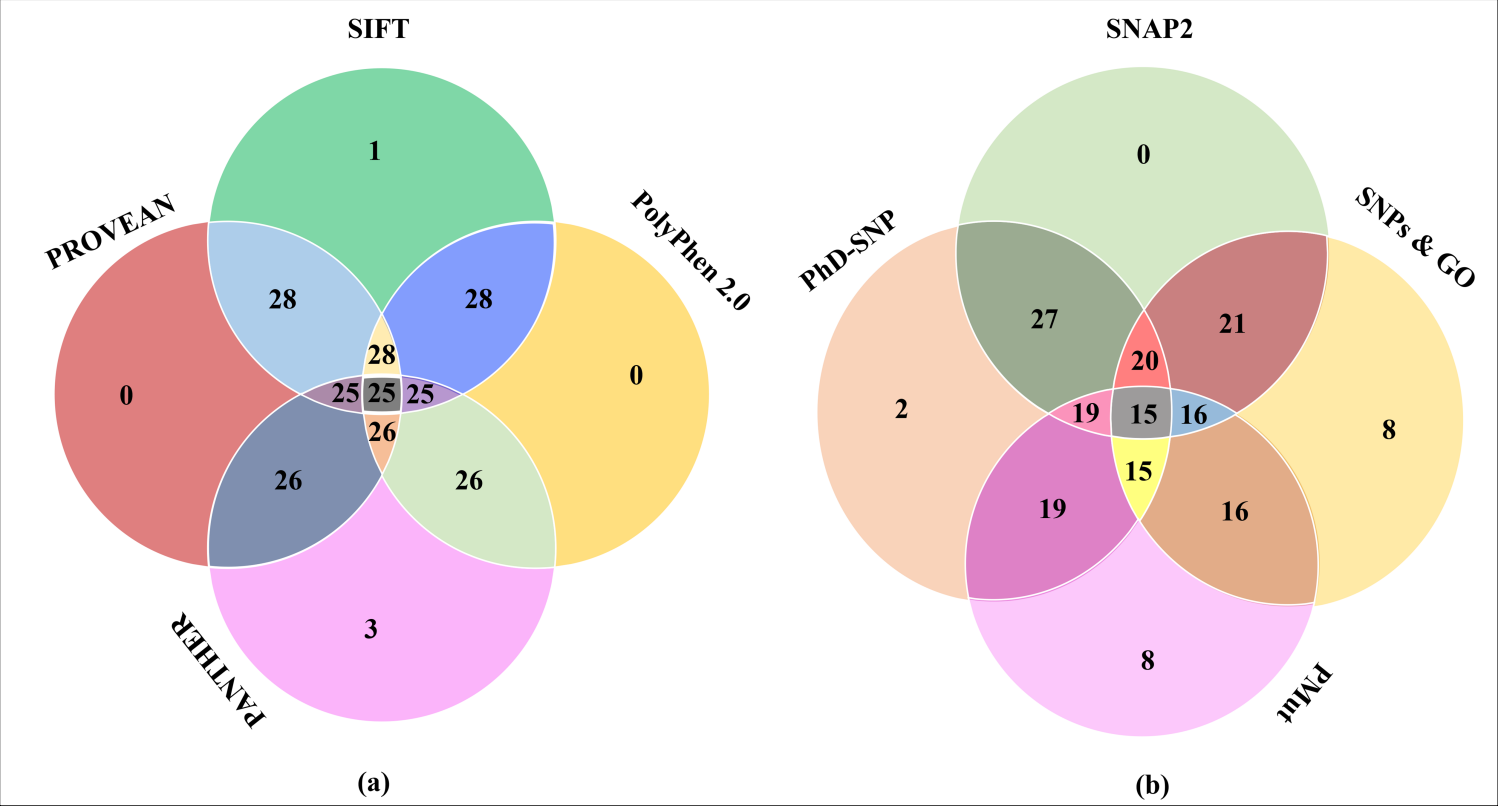
A Venn diagram representation of most deleterious nsSNPs estimated by various tools. (a) Deleterious nsSNPs identification by SIFT, PolyPhen 2.0, PROVEAN and PANTHER which causes functional alterations; (b) Disease inducing nsSNPs identification by SNAP2, PhD-SNP, SNPs and GO and Pmut)

PMut characterized 22nsSNPs to be pathological out of the given 171 mutations. Thus, 13% of the total nsSNPs were prone to giving rise to diseased state in humans. A Venn diagram is prepared distributing the nsSNPs having most damaging effect structurally-functionally according to the various in silico tools employed in this study (Figure 2). The most deleterious nsSNPs from these findings are selected for further analysis.

### Protein stability change and Prediction of conserved nsSNPs:

**Figure 3:**
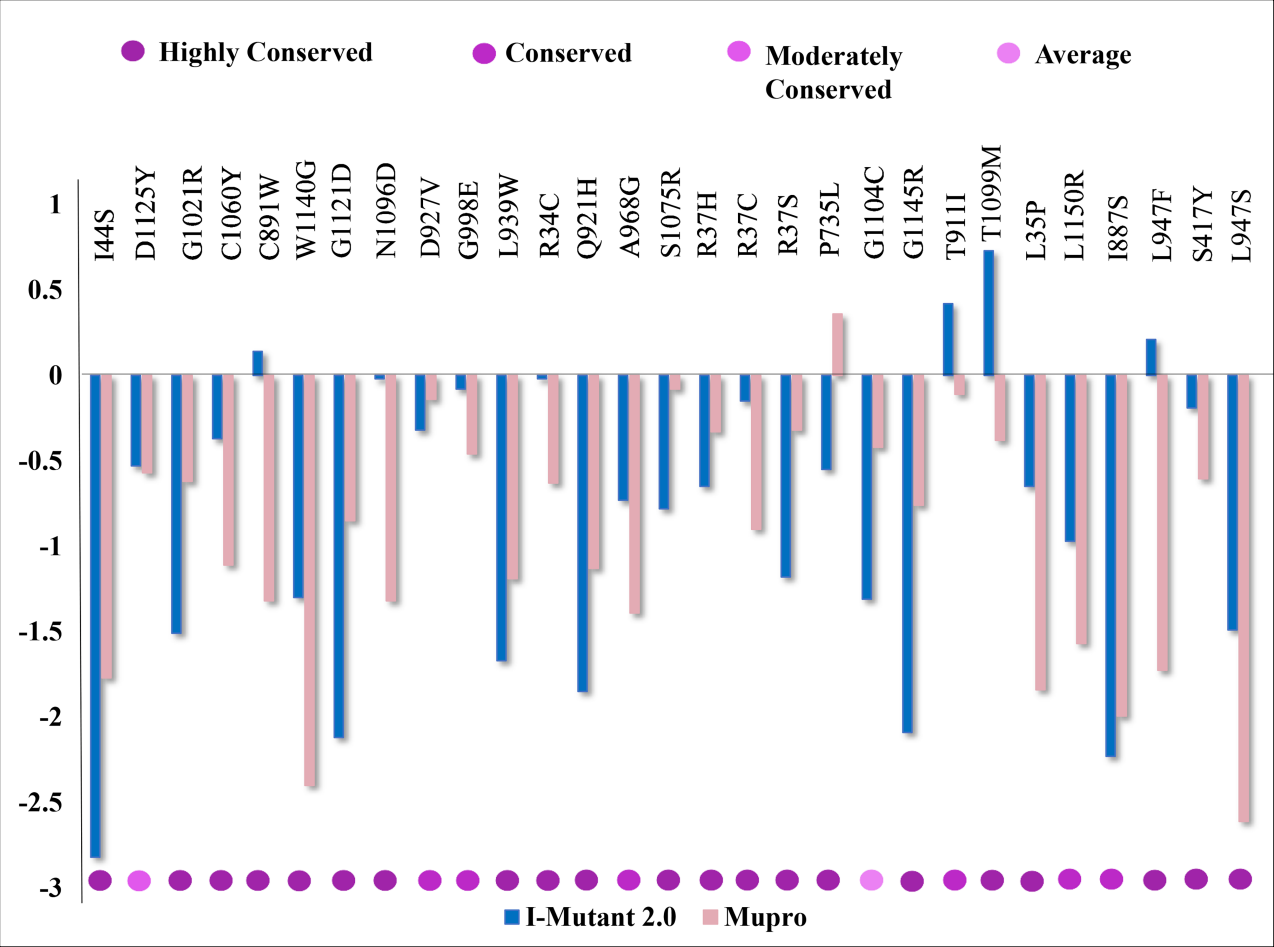
Amino acid variations distributed into conservation scale by Consurf and protein stability change prediction by I-mutant 2.0 and MUpro.

Shortlisted 29 nsSNPs were analyzed to investigate the change in protein stability. They were submitted to I-mutant 2.0 and MUpro. In both cases majority of 29 nsSNP showed destabilized effect on protein, with few increasing its stability. Among them, 22 nsSNPs belonged to C terminal domain of PALB2 protein, WD40. Conserved sequences are important in finding homology between different species and can also be used to identify the cause of genetic diseases. The evolutionary conservation pattern of amino acids or nucleotides in a protein was measured by Consurf to discover the structurally and functionally important regions [42]. From Consurf, out of 28 deleterious nsSNPs, 27 nsSNPs were found to be located on highly conserved regions with a score between 7 to 9 (Figure 3). The nsSNP in 1104 position which was moderately conserved with conservation score 6, was excluded from further analysis.

### Prediction of solvent accessibility:

NetSurf P 2.0 analyzed the secondary structure of the above-mentioned residues, categorizing them as helix, strand or coiled. 50% of these residues were exposed and 50% were buried. NetSurf P2.0 further showed that among these residues, R34, L35, R37 and P735 were moderately disordered and S417 was highly disordered (Supplementary Table 2).

### Post Translational Modification Sites Analysis:

Post translational Modification (PTM) sites were predicted via Modpred, GPS-SUMO, GPS- Palm, GPS-MSP, iGPS. PTMs with high or medium confidence were taken into analysis. Modpred predicted position R34, R37 and Q921 as proteolytic cleavage sites in wild protein. No phosphorylation and methylation sites had been predicted by iGPS and GPS-MSP. GPS-SUMO and GPS- Palm identified position N1096 as a site of sumo interaction and C1060 as a site of palmitoylation.

### Predicting structural and functional alteration:

The results obtained from MutPred2.0 revealed that 19 nsSNPs out of 27 nsSNPs had g-score greater than 0.5 with a significant p-value. These substitutions contributed to a greater risk of structural and functional alterations with significant P-value. The amino acid variations which had g-score less than 0.5, alteration probability less than 20% and p -value greater or equal than 0.05 had been excluded from the list (Table 1).

### Analysis of nsSNPs in non- coding regions: 

From Ensembl, 110 variations have been found on the non-coding regions. Out of 110 variants, 17 of them proved to be functionally important by RegulomeDB. All of them returned the score of 2(a-c) (Supplementary Table 3) where, lower the RegulomeDB score, greater the functionality. PolymiRTS outlined 3 nsSNPs rs180748355, rs185410736, rs189962793 that alter miRNA target site (Table 2).

### Structural analysis:

For structural analysis, the WD40 domain of PALB2 protein was emphasized due to lack of protein structure availability in Protein Data Bank (PDB).

### Project HOPE:

The structural effects caused by amino acid mutations such as loss of hydrogen bond formation, hydrophobicity change, disruption of protein folding etc. were explored by HOPE. The deleterious nsSNPs situated within WD40 domain were submitted to HOPE. One of the most common structural variations were the interruption of interaction with POLH (DNA polymerase eta) and POLH DNA synthesis stimulation. Mutations at protein surface area such as D1125Y, G1021R and L939W had led to disrupt the interaction with neighboring ligand molecules. Moreover, mutation at 998, were found to be associated with breast cancer susceptibility. L939W reduces interaction with BRCA2, RAD51, XRCC3 and decreases double stranded DNA break initiated homologous recombination associated with breast cancer susceptibility (Table 3).

### Mutant structures modelling:

Swiss Modeler modelled 16 mutant structures. One template with GMQE (Global Model Quality Estimation) of 0.92 was selected to build these 11 models. GMQE score of 0.92 reflected the expected accuracy of model built with that alignment. The mutant models all had a score near to zero with a “thumbs-up” beside the QMEAN score (Qualitative Model Energy Analysis) suggesting they were of good quality. Additionally, all the models built by Swiss Modeler were validated by PROCHECK, a representative of Ramachandran Plot. Each of them had more than 86% region in favored region. (Supplementary Table 4)

### RMSD, TM score and cluster prediction:

**Figure 4:**
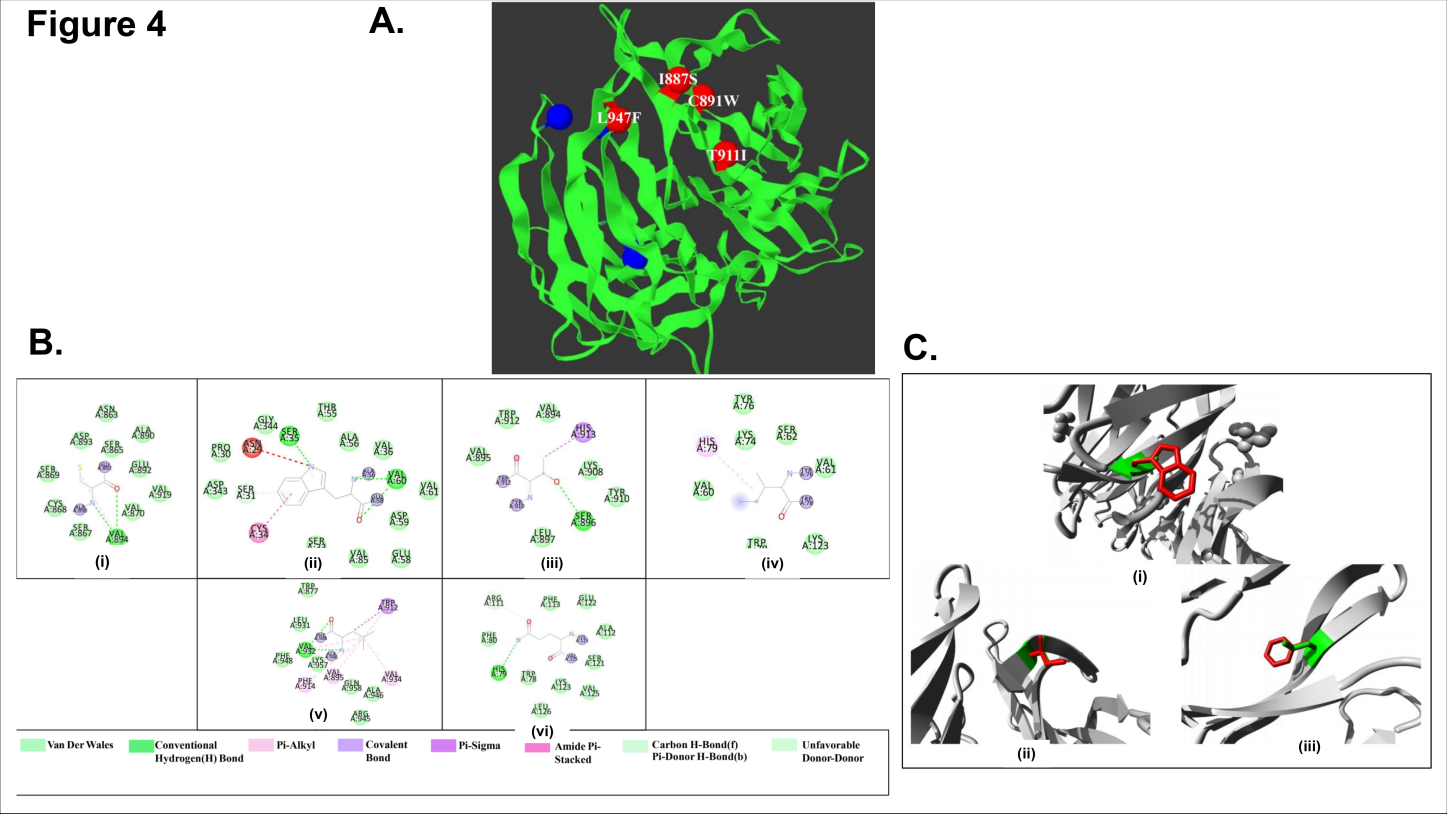
A) Visualization of cluster formation by 4 mutant models. B) Visual representation of different amino acid interactions where (i) represents Cysteine at position 891, (ii) substituted by Tryptophan at position 891. (iii) Represents Threonine at position 911, (iv) substituted by Isoleucine at position 911. (v) Represents Leucine at position 947, (vi) substituted by Phenylalanine at position 947. C) A visual representation of structural differences in residue between wild type and mutated structure. Here, (i) Represents Cysteine at wild type structure substituted by Tryptophan at position 891. (ii) Represents Threonine at wild type structure substituted by Isoleucine at position 911. (iii) Represents Leucine at wild type structure substituted by Phenylalanine at position 947.

Superimposition of wild type and mutant structures via PyMol, yields that model C891W had the highest RMSD value of 1.463 followed by several other models (D927V, D1125Y, G998E, A968G, T911I, I887S, L939W, Q921H, L1150R) with RMSD value of 1.459 and 1.458. The one with the lowest RMSD value of 1.266 was mutant model L947S. There were 3 other models (C1060Y, G1121D and W1140G), which possessed lower RMSD value close to 1.266. All the models had TM- score not greater than 0.99918. L939W, L1150R and Q921H models had the highest TM-score. Finally, based on RMSD value and TM- score prediction, 8 models (C891W, D927V, G998E, G1021R, L947F, T911I, A968G and I887S) were selected to estimate cluster formation via Mutation 3D (Supplementary Table 5). All the models submitted had greater MPQS (ModPipe Quality) score from the minimum quality requirement threshold. Mutation 3D showed that 4 models (I887S, C891W, T911I and L947F) were involved in cluster formation. All 4 models had significant p- value of 0.000504 and were pictured as red cluster balls (Figure 4A).

### Difference in amino acid interaction:

Out of the 4 models previously mentioned, 3 mutant models (C891W, T911I and L947F) had been selected for further analysis via Discovery Studio Visualize as they provoke the most structural alterations. The amino acid interactions at different positions are shown both in case of native structure and mutant structures (Figure 4B). Upon mutation, each of the native models interacted differently than wild type.

## Discussion:

**Figure 5:**
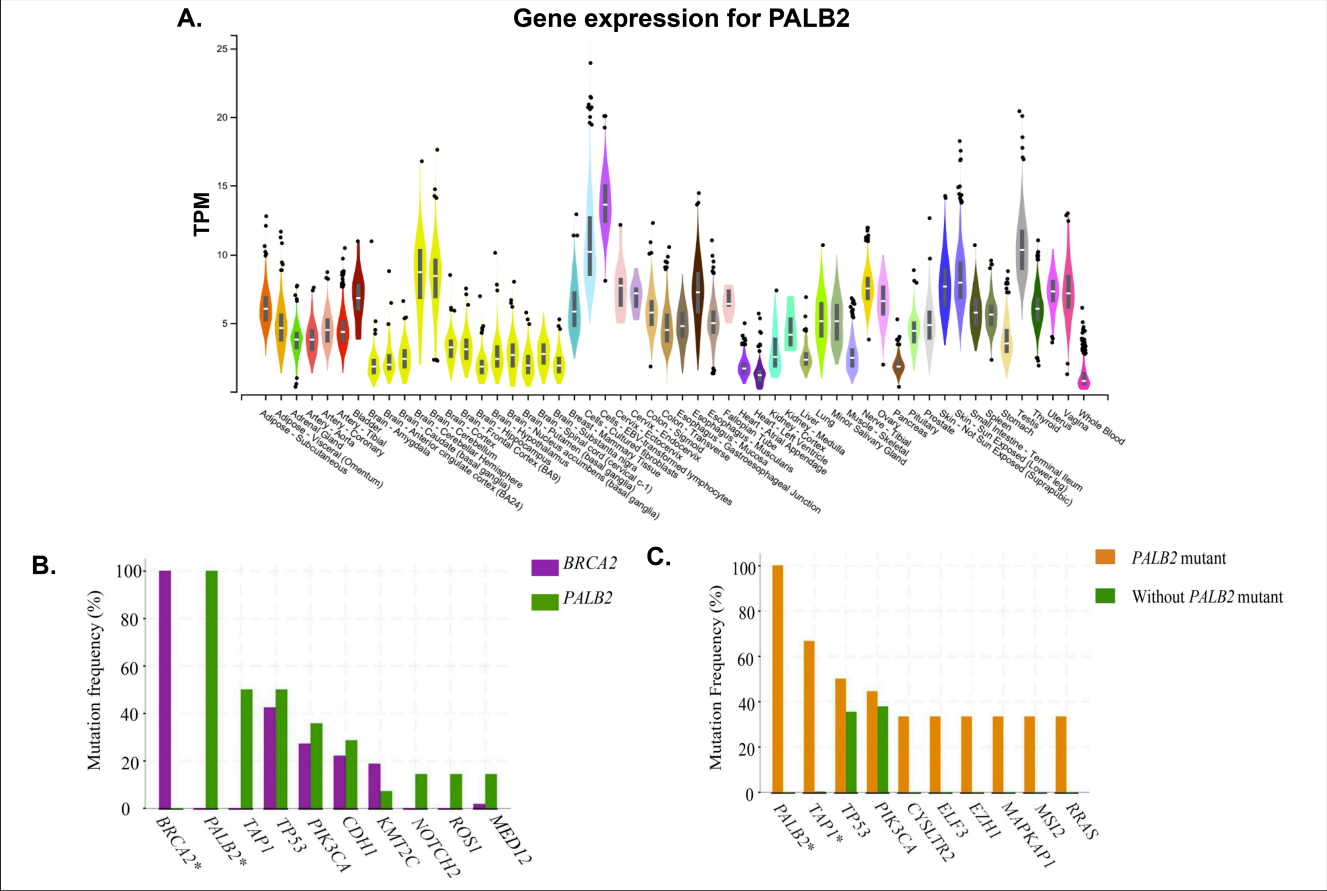
A) Gene expression of PALB2 in specific tissues. B) Comparison of BRCA2 and PALB2 mutation frequency. C) Comparison of mutation frequency with PALB2 mutation carriers and without PALB2 mutation carriers.

Most human non-synonymous single nucleotide polymorphisms (nsSNPs) represent genetic variations along with phenotypic differences. The goal of nsSNP research is to comprehend its association with many complex human diseases in genetic level [43]. PALB2 (partner and localizer of BRCA2) binds with BRCA2 (breast cancer 2) in nuclear foci and thus permits stable intra-nuclear localization and accumulation of BRCA2. The interaction between PALB2 and BRCA2 is important for maintaining genomic integrity [44]. Moreover, BRCA2 and BRCA1 are the most common causes of hereditary breast cancer [45]. Loss of function mutations in PALB2 can also lead to hereditary breast cancer [46]. Thus, the current computational analysis has been done to point out the single amino acid variations responsible for alteration of functional and structural attributes of PALB2. 1454 missense variants on the protein coding region of PALB2 were collected from NCBI dbSNP database and submitted into various functional alteration predicting tools such as SIFT, PolyPhen, PROVEAN, SNAP2, PhD-SNP, PANTHER, SNPs and GO and PMut in order to reveal the most deleterious mutations. After that, I-Mutant 2 and MUpro to find out their capability to stabilize or destabilize the PALB2 examined 28 nsSNPs. In addition, Consurf web server predicted 27 out of 28 nsSNPs to be highly conserved. Location of those amino acid variations on protein surface or protein center was also marked via Netsurf P2.0. Further, post translational modification (PTM) sites have also been predicted. R37 and Q921 positions were identified as a site of proteolytic cleavage. Position N1096 was found as a site of Small Ubiquitin-like Modifiers (SUMO) interaction that may alter the ubiquitin binding. And position C1060 was found to be a site of palmitoylation, where fatty acids like palmitic acid are covalently attached to cysteine. Alteration of amino acid residues on these positions due to SNPs will affect the modification, which may cause loss of functionality in the protein. The rest of the nsSNPs did not contain any of the PTMs. In subsequent steps, using MutPred2.0, 19 nsSNPs among the 27s were found to have the harmful structural and functional alterations on the protein with a high g -score (>0.75). In addition to that, structural impact of these nsSNPs located within PDB available C terminal WD40 domain of PALB2 was observed. This analysis was particularly done, since mutations in WD40 Domain leads to cancer formation [47].WD40 domain included residues from 835-1186 where 16 mutant models were built using SWISS-Model and further were examined by Project HOPE for structural analysis. For further interpretations, the mutants RMSD value and TM score was compared. The higher the RMSD value and the lower the TM-score, the diverse the mutant structures were from the native structure. Most of the mutant models had RMSD score near 1.459 and TM score near 0.99915. Few mutant models which achieved low RMSD score around or less than 1.266 and high TM score greater than 0.99915 were excluded from later analysis. Thus 9 mutant models (C891W, D927V, D1125Y, G998E, G1021R, L947F, T911I, A968G, I887S) were chosen to be submitted into Mutation 3D web server for the identification of the clusters of mutation on the protein structure. Out of 9 models only 4 (I887S, C891W, T911I and L947F) of them formed cluster. Mutation cluster mainly provides a data of single residue mutational hotspot across various cancer types [48]. These can eventually bring about functional driver genes. Finally, 3 models (C891W, T911I and L947F) were chosen based on their RMSD value, TM score and cluster formation data. A visual representation of C891W, T911I and L947F mutation models by HOPE depicted bigger amino acids in structure and thus do not fit into the core of protein (Figure 4C). Besides, charged mutations cause repulsion between mutant and neighboring residues. Difference in the interaction among amino acids from the wild type has been shown via discovery studio visualizer. In case of C891W, the mutant structure attained one pi-donor hydrogen bond with Serine. It also interacted with Cysteine via amide pi- stacking. In T911I mutant model, the mutant structure lost a pi sigma bond with Histidine and gained a pi- alkyl bond instead. Besides that, hydrogen bond with Serine got disrupted and a pi-alkyl interaction with histidine was observed. Lastly, the third mutant model, L947F, when Phenylalanine substituted Leucine, interaction with Tryptophan via pi-sigma bond and alkyl bonds with valine and phenylalanine got interrupted. Instead, a new C-H bond was created, with Arginine. Those intra-molecular bonds are crucial for protein structure as well as function. However, most of the human genome consist of non-coding regions and single nucleotide polymorphism in this region can affect gene expression pattern, gene splicing transcription factor binding, miRNA target site binding etc. [49]. Thus, nsSNPs in non-coding region can also be pathogenic and can manifest into a higher risk of cancer. Therefore, non-coding nsSNPs, retrieved from Ensemble were analyzed via RegulomeDB. It examines the putative function of the genetic variants to identify a significant association among multiple tagged SNPs in complex diseases [50]. 17 mutations were found to be pathogenic and according to PolymiRTS, three mutations altered the targeted miRNA binding sites. SNPs located within or near the regions that are required for interaction with other proteins can alter the protein interaction complexes altogether [51]. Based on STRING, PALB2 closely interacts with BRCA2, BRCA1, RAD51, RAD51C and FANCD2. PALB2 serves as the molecular scaffold for the formation of BRCA2-PALB2-BRCA1 complex through its ability to recruit BRCA2 and RAD51. FANCD2 is also involved in DNA double stranded break repair by homologous recombination [52]. Thus, mutation in any functionally important region in PALB2 can lead to interruption of DNA repair mechanism and eventually cause disruption in genomic integrity. According to GWAS (Genome Wide Association Study) catalog, PALB2 gene has been seen associated with bipolar disorder [53]. Moreover, from GTEx portal it was found that PALB2 is maximally expressed in EBV transformed lymphocytes and fibroblasts and minimally expressed in whole blood (Figure 5A). It is also fairly expressed in breast mammary tissues, brain cerebellum and cerebral hemisphere. Elevated chromosome instability is observed in lymphocytes with PALB2 mutation carriers [54]. PALB2 mutations may have a higher risk of breast cancer predisposition than BRCA2 variants [55] (Figure 5B). Besides, in the germline mutation analysis, it was clearly seen that somatic gene mutation rate of other genes that are responsible for breast cancer are much higher in case of PALB2 mutation (Figure 5C). Thus, this mutation analysis depicts the importance of PALB2 mutation studying biological correlation in various disease conditions.

## Conclusion

In this study, non- synonymous single nucleotide polymorphisms (nsSNPs) were characterized that alter PALB2 protein structure and functional activity. Out of initially screened 28 deleterious nsSNPs, the most damaging nsSNPs were identified. Due to unavailability of whole protein structure of PALB2 protein in Protein Data Bank, only nsSNPs within WD40 domain (PDB code: 2W18) were emphasized for structural investigations. Lastly, 3 mutations were chosen to be most deleterious according to RMSD and TM score. Importance of PALB 2 investigated from cBioportal, GTEx and GWAS portal also indicate the pathogenic effect of deleterious SNPs. Thus, this study provides us with a new region to look into diseases like breast cancer and investigate the proteins mechanism performing further polymorphism analysis studies.

## Figures and Tables

**Table 1:** Effect of nsSNPs on the structure and function of protein predicted by MutPred 2.0.

SNP ID	Mutant	MutPred2.0 score	Impact and probability	P-value
rs78179744	C891W	0.786	Gain of strand (30%)	0.003
			Altered transmembrane protein (22%)	0.003
			Altered ordered interface (25%)	0.02
rs116967702	C1060Y	0.871	Altered ordered interface (34%)	0.001
			Altered metal binding (23%)	0.04
rs62625280	D927V	0.886	Altered transmembrane protein (42%)	0.000005
			Loss of loop (29%)	0.01
			Altered ordered surface (26%)	0.01
rs146444298	D1125Y	0.81	Altered metal binding (55%)	0.003
			Altered ordered surface (41%)	0.0003
rs45551636	G998E	0.665	Gain of intrinsic disorder (31%)	0.04
			Loss of B-factor (26%)	0.04
rs143808171	G1021R	0.8	Gain of helix (29%)	0.01
			Altered transmembrane protein (26%)	0.001
rs62625282	G1121D	0.857	Altered metal binding (39%)	0.009
rs45478192	L939W	0.862	Gain of loop (27%)	0.03
			Altered transmembrane protein (22%)	0.004
rs62625283	W1140G	0.942	Altered ordered surface (48%)	0.0003
			Altered metal binding (22%)	0.02
rs45464500	L947S	0.821	Altered stability (80%)	0.0003
			Gain of intrinsic disorder (46%)	0.03
rs141047069	L35P	0.848	Altered coiled coil (90%)	0.0007
			Altered disordered interface (42%)	0.0006
			Loss of helix (28%)	0.02
			Loss of acetylation at K30 (26%)	0.01
rs45566737	L1150R	0.811	Gain of strand (27%)	0.03
			Altered transmembrane protein (21%)	0.004
				
rs45510998	S417Y	0.814	Altered ordered interface (30%)	0.02
			Altered disordered interface (30%)	0.02
			Altered DNA binding (25%)	0.009
			Gain of allosteric site (23%)	0.02
rs45550935	I887S	0.799	Gain of strand (27%)	0.02
			Altered stability (20%)	0.01
rs372931676	Q921H	0.717	Altered transmembrane protein (30%)	0.0002
rs200048921	R37S	0.622	Altered disordered interface (47%)	0.004
			Altered DNA binding (23%)	0.01
rs369132015	A968G	0.526	Gain of strand (26%)	0.04
rs180177123	T911I	0.534	Altered transmembrane protein (33%)	0.00006
			Altered ordered interface (26%)	0.01
rs45525135	L947F	0.655	Altered stability (25%)	0.008

**Table 2:** Predicted results of noncoding SNPs in miRNA target site. MiRSite: sequence context of the miRNA site: bases complementary to the seed region are in capital letters and SNPs are highlighted in bold form; Function class: D= the derived allele disrupts a conserved miRNA site (ancestral allele with support>2); C= the derived allele creates a new miRNA site; N= the derived allele disrupts a non- conserved miRNA site (ancestral allele with support<2); Context score= negative increase= increase of SNP functionality.

SL no.	SNP ID	Allele	miRID	miRSite	Function class	Context+score change
1	rs180748355	G	hsa-let-7c-3p	acatTTGTACAtg	D	-0.148
		T	hsa-miR-4495	acattTTTACATg	C	-0.08
			hsa-miR-548c-3p	acATTTTTAcatg	C	0.069
2	rs185410736	A	hsa-miR-1250-3p	acacttAAAATGA	D	-0.002
			hsa-miR-153-5p	acacttAAAATGA	D	0.015
			hsa-miR-595	ACACTTAaaatga	N	-0.13
			hsa-miR-7856-5p	acaCTTAAAAtga	N	-0.001
		C	hsa-miR-3616-5p	aCACTTCAAatga	C	-0.387
			hsa-miR-4795-5p	aCACTTCAaatga	C	-0.14
			hsa-miR-573	aCACTTCAAatga	C	-0.345
			hsa-miR-579-3p	acacttCAAATGA	C	-0.116
			has-miR-664b-3p	acacttCAAATGA	C	-0.125
3		G	hsa-miR-4279	tataaAGGAGAAt	D	-0.169
	rs189962793	A	hsa-miR-130b-5p	tataAAAGAGAat	C	-0.098
			hsa-miR-4753-3p	tataAAAGAGAAt	C	-0.313
			hsa-miR-6809-3p	tataaAAGAGAA	C	-0.2

**Table 3:** Structural consequences prediction of nsSNPs by Project HOPE.

Mutant Models	Difference in size	Hydrophobicity	Charge Change	Alteration in bond formation
C891W	Bigger	-	-	-
C1060Y	Bigger	Decreased	-	-
D927V	Smaller	Increased	Negative to neutral	Disrupts H-bond formation with Lys at position 974;
				Disrupts salt bridge formation with Arginine at position 975 and position 976
D1125Y	Bigger	Increased	Negative to neutral	Disrupts salt bridge formation with lysine at position 1062 and position 1124
G998E	Bigger	Decreased	Neutral to negative	-
G1021R	Bigger	Decreased	Neutral to positive	Disrupts local structure backbone
G1121D	Bigger	Decreased	Neutral to negative	-
L939W	Bigger	-	-	-
W1140G and A968G	Smaller	Decreased	-	W1140G disrupts hydrogen bond formation with Cysteine at position 1109
L947F and Q921H	Bigger	-	-	-
L1150R	Bigger	Decreased	Neutral to positive	-
L947s and I887S	Smaller	Decreased	-	-
T911I	Bigger	Increased	-	Loss of H-bond;
				Disrupts correct folding
